# Allergic sensitization in the tropics: unravelling co-sensitization between crustacean and mites

**DOI:** 10.3389/falgy.2025.1674423

**Published:** 2025-11-14

**Authors:** Sahel Heidari, Shaymaviswanathan Karnaneedi, Elecia Johnston, Emily Jerry, Diamond Hira, Andreas Ludwig Lopata

**Affiliations:** 1 Molecular Allergy Research Laboratory, College of Science and Engineering, James Cook University, Townsville, QLD, Australia; 2 Australian Institute of Tropical Health and Medicine, James Cook University, Townsville, QLD, Australia; 3 Centre for Food and Allergy Research, Murdoch Children’s Research Institute, Melbourne, VIC, Australia; 4 Allergy Medical Centre, Mundingburra, QLD, Australia; 5 Tropical Futures Institute, James Cook University Singapore, Singapore, Singapore

**Keywords:** crustacean allergy, mite allergy, tropomyosin, tropics, ALEX2

## Abstract

Crustacean allergy, a common food allergy triggered by immune reactions to proteins in shrimp, lobster, and crab, involves over ten identified allergenic proteins and can lead to severe symptoms, including anaphylaxis. Shellfish-allergic individuals are often sensitized to house dust mites (HDM), yet the specific interrelationships remain unclear. This study analyzed 93 seafood-allergic individuals and selected 54 subjects with positive skin prick tests and/or ImmunoCAP-measured specific IgE to crustaceans. Allergy Explorer 2 (ALEX2) testing was subsequently performed for these individuals. The most reactive mite allergens (in total over 81%), included Der p 1, Der p 2, Der p 23, Der f 1, Der f 2, Gly d 2, and Lep d 2. Der p 23 alone was identified in over 61% of crustacean allergic subjects. Anaphylaxis was reported in 11 subjects, correlating strongly with IgE sensitization to tropomyosin (63.6%), but also to Lep d 2 and Der p 23 (both 54.5%). These findings stress the complex sensitization patterns in crustacean-mite allergic individuals in the tropics, highlighting both tropomyosin and non-tropomyosin cross- and co-reactivity. The significant IgE reactivity to Der p 23 and arginine kinase suggests the need for enhanced diagnostic approaches and further investigation into the clinical implications of these sensitization patterns in crustacean and mite sensitised individuals for improved allergy management.

## 1 Introduction

Shellfish allergy is a significant cause of food hypersensitivity, affecting approximately 5 to 16% of both adults and children globally (1). It is also the leading cause of anaphylaxis in tropical regions (1). This allergy is often lifelong, with up to 90% of affected individuals retaining their sensitivity into adulthood (2). Shellfish allergy is one of the predominant causes of food-induced anaphylaxis in several countries, including Australia, Singapore, Hong Kong, Korea, Mexico, Pakistan, Philippines, Portugal, Russia, Taiwan, Thailand, Tunisia, and Italy (3–13). Of the hundreds of shellfish species consumed globally, shrimp seems to be the most common trigger for allergic reactions worldwide (14).

In contrast to other major food allergies, the clinically relevant allergens involved in shrimp allergy have not been thoroughly characterized across most populations, with notable variability in sensitization profiles between individuals residing in temperate and tropical regions. Shrimp allergy presents with diverse clinical phenotypes, ranging from oral allergy syndrome with mild oral symptoms to severe reactions (15), including anaphylaxis often triggered by minimal cross-contamination. Symptoms are primarily mediated by IgE antibodies, making the understanding of the mechanisms underlying sensitization, particularly cross-reactivity with other allergens, critical for improving diagnosis and management strategies (14).

The key allergens responsible for shellfish allergies are proteins including tropomyosin, arginine kinase, while in total eleven allergens are registered with the IUIS (13, 16). The most comprehensive research into crustacean allergens has occurred in shrimp species such as black tiger shrimp *(Penaeus monodon)* and white leg shrimp (*Litopenaeus vannamei)*, due to their widespread consumption globally (17). Tropomyosin, one of the most extensively studied shellfish allergens, is recognized as a highly cross-reactive food allergen, with cross-reactivity also observed with group 10 allergens, the tropomyosin from mites (18–20). This cross-reactivity is likely due to the IgE antibody recognition of structural and amino acid homologies between these allergens. Other potential cross-reactive allergens, such as arginine kinase and hemocyanin, as well as minor mite allergens homologous to shrimp allergens, are also of potential clinical interest (21, 22).

Previous prospective studies have indicated that early sensitization to house dust mites may increase the risk of developing shellfish sensitization (23, 24). Given the high prevalence of adult shellfish allergy in many countries (25), understanding the co-sensitization profiles to various dust mite allergens in individuals sensitized to shellfish is crucial for optimal patient management and therapeutic interventions.

With the expanding global data on the rising IgE sensitization to different indoor and storage mite species and their allergens, the current study examines the sensitization profiles of a clinically confirmed shellfish-allergic individuals against 17 individual mite allergens and two whole mite extracts.

## 2 Materials and methods

### 2.1 Study population

As part of Australia's largest cohort study on crustacean allergy, 93 seafood-allergic patients were initially recruited through a specialist allergy clinic servicing the tropical regions of Queensland, Australia. Eligibility required a documented clinical history of immediate allergic reactions to seafood, confirmed by an allergy specialist and recorded in medical files. From this group, 54 individuals were selected based on a well-documented history of crustacean allergy and a positive skin prick test (SPT) and/or ImmunoCAP test for crustaceans. For analyses requiring detailed symptom classification, only 42 subjects were included, as clinical information regarding symptom type or organ involvement was incomplete for 12 individuals. The cohort comprised both paediatric (<18 years) and adult participants, with demographic details provided in Table 1.

**Table 1:** Demographics and diagnostic results of 54 subjects.

Type of patient	Paediatric % (*n*)	33.3% (18)
	Adult % (*n*)	66.6% (36)
Age	Median (Min-Max)	28.5 (9–73)
Mite ALEX	Pos % (*n*)	85.2% (46)
	Neg % (*n*)	14.8% (8)
HDM Immunotherapy	Yes % (*n*)	29.6% (16)
	No % (*n*)	70.3% (38)
Gender	Female % (*n*)	55.5% (30)
	Male % (*n*)	44.4% (24)
Crustacean ImmunoCAP (prawn, crab)	Pos % (*n*)	77.7% (42)
	ND % (*n*)	5.5% (3)
	Neg % (*n*)	16.6% (9)
	Mean IgE (min-max) (kU/L)	3.86 (0 ≥ 100)
Crustacean SPT (raw/cooked prawn and crab)	Pos % (*n*)	90.7% (49)
	ND % (*n*)	7.4% (4)
	Neg % (*n*)	1.8% (1)
	Mean wheal size (min-max) (mm)	4.48 (0–20)

Blood samples were collected in EDTA-containing tubes, followed by centrifugation at 3,000 rpm for 10 min at 4 °C. The resulting plasma was stored at −80 °C for subsequent analysis. Ethical approval was granted by the James Cook University Ethics Committee (Project numbers: H4313, H6829). All participants received detailed study information and provided written informed consent before enrolment.

### 2.2 Clinical diagnostic tests: SPT and ImmunoCAP

SPT was conducted using commercially available crustacean extracts, including prawn (*Litopenaeus setiferus*, *Farfantepenaeus aztecus*, and *Farfantepenaeus dourarum*), crab (*Callinectes* spp.), codfish (*Gadus* spp.), and lobster (*Homarus americanus*) (ALK-Abelló, Madrid, Spain). Histamine (10 mg/mL) served as the positive control, while normal saline was used as the negative control. Wheal diameters were assessed 15 min post-application, with a diameter ≥3 mm considered a positive reaction.

Specific IgE (sIgE) levels were measured using ImmunoCAP assays (Thermo Fisher, USA), with a cutoff of ≥0.35 kU/L applied for positivity. The ImmunoCAP panel included crustacean allergen components to establish a comprehensive sensitization profile. This panel quantified IgE reactivity to f23 (crab; *Chionoecetes spp.*), f24 (shrimp mix; including *Pandalus borealis*, *Penaeus monodon*; *Metapenaeopsis barbata*, and *Metapenaeus joyneri*), f80 (lobster; *Homarus gammarus*), and f320 (crayfish; *Astacus astacus*).

### 2.3 Multiplex IgE assay (ALEX_2_ macroarray)

The Allergy Explorer 2 (ALEX_2_) macroarray assay (Macro Array Diagnostics, Vienna, Austria) was employed to measure sIgE reactivity to 117 allergen extracts and 178 purified allergen components, providing a comprehensive assessment of crustacean and mite sensitization profiles.

The panel included a broad range of crustacean allergen extracts, such as Chi spp., Hom g, Lit s, Far a, Far d, Pan b, but also individual crustacean allergens; Pen m 1, Pen m 2, Pen m 3, Pen m 4, and Cra c 6. In addition mite allergens analyzed in this assay comprised of Der p 1, Der p 2, Der p 5, Der p 7, Der p 10, Der p 11, Der p 20, Der p 21, Der p 23, Der f 1, Der f 2, Blo t 5, Blo t 10, Blo t 21, Gly d 2, Lep d 2, Tyr p 2 and whole allergen extracts of Tyr p, and Aca s. A comprehensive list of allergens is provided in Supplementary Table S1. The ALEX_2_ immunoassay utilizes a chip-based technology incorporating a nitrocellulose membrane for broad-spectrum allergen profiling. This assay includes a cross-reactive carbohydrate determinant (CCD) inhibitor within the serum diluent to eliminate potential CCD interference. Following an additional washing cycle, an enzyme substrate was applied, and the reaction was completed within eight minutes. A cutoff of ≥0.35 kU/L was used to determine positivity.

### 2.4 Statistical analysis

All statistical analyses were conducted using GraphPad Prism version 9.0, while both GraphPad Prism and Microsoft Excel were used for data visualization. As the data did not follow a normal distribution, the Mann–Whitney U test was used to compare IgE levels between groups. A *p*-value <0.05 was considered statistically significant.

## 3 Results

### 3.1 Sensitization to mite allergens

**Figure 1:**
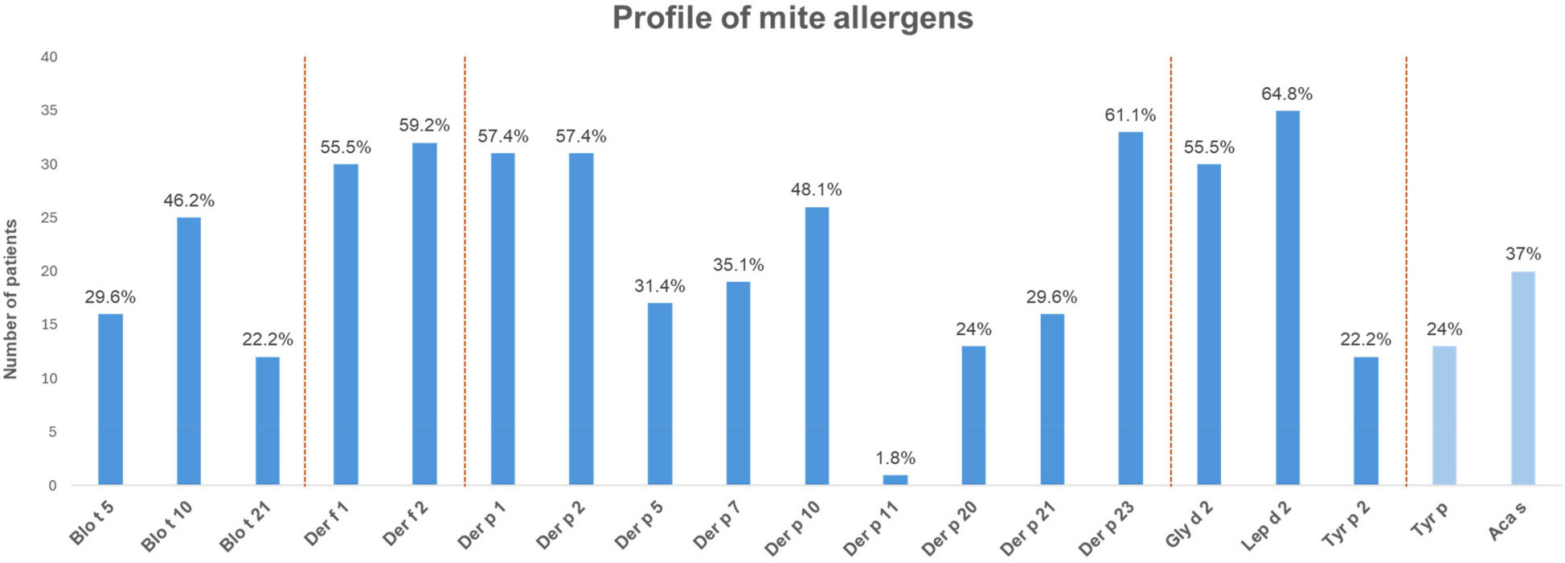
Frequency of positive IgE responses to mite allergens in 54 crustacean-allergic subjects. Dark blue bars indicate sensitization to mite single components, whereas light blue bars represent sensitization to mite whole extract. Percentages above each bar indicate the proportion of subjects sensitized to the corresponding allergen. A specific IgE result of ≥0.35 kU/L was considered positive.

The sensitization profile of 54 crustacean-allergic subjects to various mite allergens is illustrated in Figure 1 and Supplementary Figure S1. Among the tested allergens, IgE reactivity was highest for Lep d 2 (*n* = 35, 64.8%) and Der p 23 (*n* = 33, 61.1%). Similarly, strong IgE binding was observed for Der f 2 (*n* = 32, 59.2%) and Der f 1 (*n* = 30, 55.5%), as well as for both Der p 1 and Der p 2 (*n* = 31, 57.4%), highlighting their roles as key contributors to mite sensitization. Sensitization to *Blomia tropicalis* (Blo t) allergens was moderate, with Blo t 10 (*n* = 25, 46.2%) exhibiting the highest IgE reactivity within this group. In contrast, lower IgE recognition was detected for Der p 5 (*n* = 17, 31.4%) and Der p 7 (*n* = 19, 35.1%), while Der p 11 (*n* = 1, 1.8%) demonstrated the least reactivity, indicating a minor role in mite allergenicity. Whole extract responses to *Acarus siro* (Aca s) (*n* = 20, 37.0%) and *Tyrophagus putrescentiae* (Tyr p) (*n* = 13, 24.0%) were comparatively lower than those observed for single components (Figure 1 and Supplementary Figure S1).

### 3.2 IgE reactivity to mite allergens in crustacean-allergic subjects based on Pen m 1 sensitization

**Figure 2:**
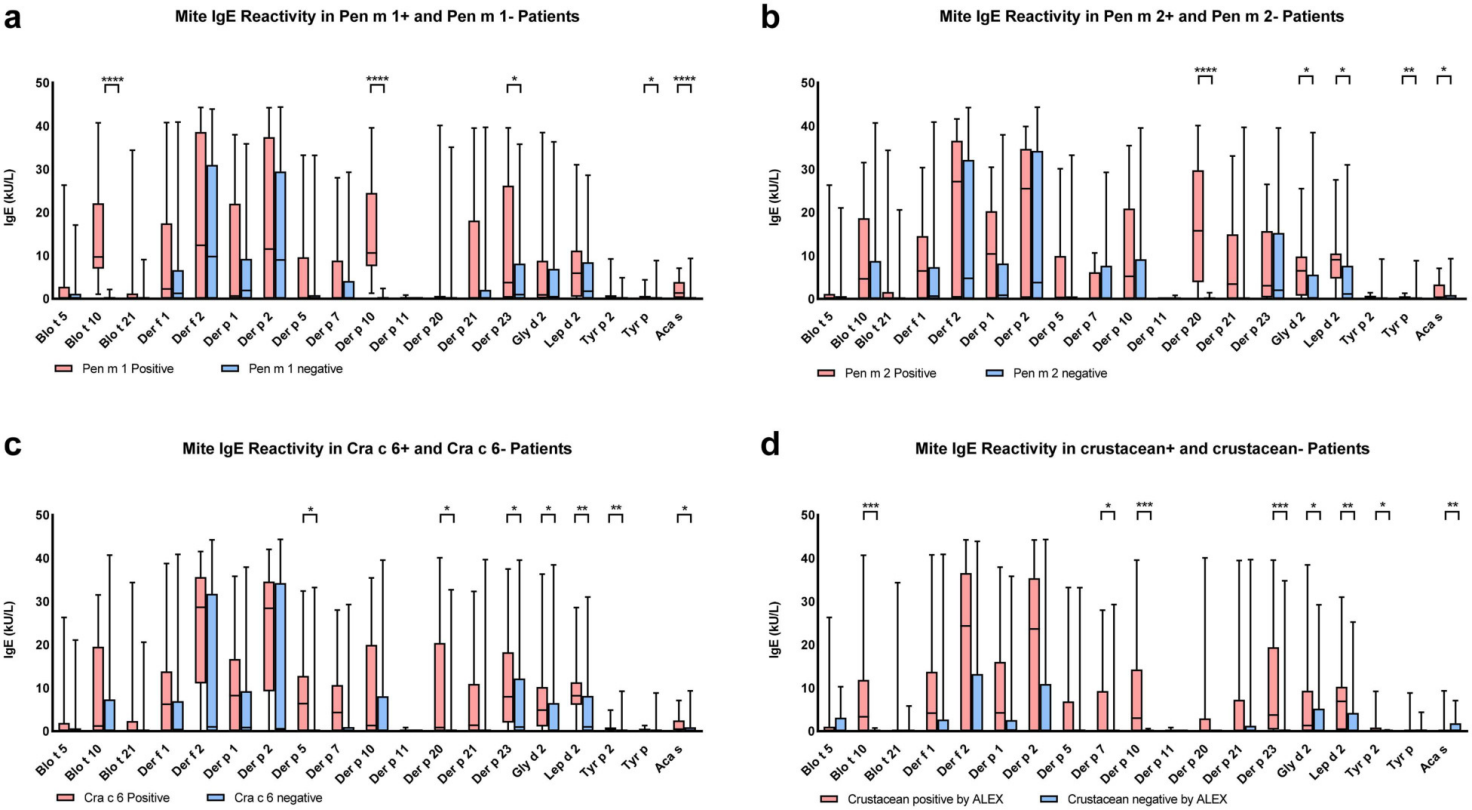
Comparison of mite-specific IgE levels (kU/L) between different subject subgroups among the 54 crustacean-allergic individuals. Each panel presents IgE responses to mite allergens, with subjects divided into two groups based on their sensitization status to specific allergens. **(a)** Mite IgE reactivity in subjects positive (pink, *n* = 22) and negative (blue, *n* = 32) for Pen m 1. **(b)** Mite IgE reactivity in subjects positive (pink, *n* = 12) and negative (blue, *n* = 42) for Pen m 2. **(c)** Mite IgE reactivity in subjects positive (pink, *n* = 14) and negative (blue, *n* = 40) for Cra c 6. **(d)** Mite IgE reactivity in subjects positive (pink, *n* = 40) and negative (blue, *n* = 14) for crustacean allergens based on ALEX testing. Statistical significance is indicated above the comparisons (*p* < 0.05: *, *p* < 0.01: **, *p* < 0.001: ***, *p* < 0.0001: ****).

The analysis of mite-specific IgE reactivity in crustacean-allergic subjects demonstrated distinct sensitization patterns based on Pen m 1 (tropomyosin) sensitisation status. Subjects sensitized to Pen m 1 (*n* = 22) exhibited significantly higher IgE levels to Blo t 10 and Der p 10, both mite tropomyosins, compared to Pen m 1-negative individuals (*n* = 32) (*p* < 0.0001) (Figure 2a).

Beyond tropomyosin, IgE reactivity to Der p 23 was significantly elevated in Pen m 1-positive subjects (*p* < 0.05), suggesting the involvement of additional co-relative components. Whole mite extract responses also differed between groups, with Aca s exhibiting a highly significant difference (*p* < 0.0001), while Tyr p showed a moderate but notable difference (*p* < 0.05) (Figure 2a).

### 3.3 IgE reactivity to mite allergens in crustacean-allergic subjects based on Pen m 2 sensitization

Subjects sensitized to Pen m 2 (arginine kinase) (*n* = 12) exhibited significantly elevated IgE levels to Der p 20 (*p* < 0.0001), supporting potential cross-reactivity between arginine kinase from crustaceans and mites (Figure 2b). Additionally, IgE levels were higher in Pen m 2-positive subjects for Gly d 2 (*p* < 0.05) and Lep d 2 (*p* < 0.05) (Figure 2b).

Although Der f 2 and Der p 2 demonstrated higher IgE binding in the Pen m 2-positive group, the differences were not statistically significant, indicating variable IgE recognition patterns. Whole mite extract responses also differed between groups, with Aca s showing significant difference (*p* < 0.05), while Tyr p exhibited a more pronounced difference (*p* < 0.01) (Figure 2b), reinforcing the complexity of mite and crustacean allergen cross-reactivity.

### 3.4 IgE reactivity to mite allergens in crustacean-allergic subjects based on Cra c 6 sensitization

Subjects sensitized to Cra c 6 (troponin C) (*n* = 14) exhibited increased IgE reactivity to Der p 5, Der p 20, Der p 23, and Gly d 2 (*p* < 0.05), as well as Lep d 2 and Tyr p 2 (*p* < 0.01). Additionally, the whole extract Aca s exhibited a significant difference (*p* < 0.05) (Figure 2c).

### 3.5 IgE reactivity to mite allergens in crustacean-allergic subjects based on crustacean sensitization

The comparison of subjects with positive IgE responses to crustaceans in ALEX2 (*n* = 40) and those negative for all single components and whole extracts in ALEX2, despite SPT and/or ImmunoCAP positivity (*n* = 14), revealed significant differences in mite-specific IgE reactivity.

Subjects sensitized to crustaceans in ALEX2 exhibited significantly higher IgE levels to Blo t 10 (*p* < 0.001), Der p 10 (*p* < 0.001), Der p 7 (*p* < 0.05), Der p 23 (*p* < 0.001), Gly d 2 (*p* < 0.05), Lep d 2 (*p* < 0.01), and the whole extracts Tyr p (*p* < 0.05), and Aca s (*p* < 0.01) compared to the ALEX2 crustacean-negative group (Figure 2d).

Within the crustacean-negative group, however, several mite allergens still showed notable reactivity, particularly Der p 23, Lep d 2, and Tyr p 2, suggesting that polysensitization to mites occurred even in the absence of identifiable crustacean component reactivity.

These findings suggest that ALEX2-negative subjects may exhibit IgE responses to unmeasured or unique allergenic determinants. As most of these allergens were tested as standardised single components, variability in extract content is unlikely to fully explain the observed differences, though it may contribute to findings involving whole extract allergens.

Within the crustacean-negative subgroup (*n* = 14), sensitization was largely confined to major HDM allergens, with Der p 1 (50%) and Der p 2 (42.9%) showing the highest frequencies. In contrast, responses to Der p 10 (14.3%) and Der p 23 (21.4%) were less common, and Der p 20 showed no reactivity, underscoring the heterogeneous and selective nature of mite allergen recognition in this cohort (Supplementary Table S2).

### 3.6 IgE reactivity to mite allergens in crustacean-allergic subjects based on HDM immunotherapy

Comparison of subjects with and without a history of HDM immunotherapy (*n* = 16 vs. 38) showed that sIgE levels were significantly higher in the immunotherapy group for Blo t 5, Der f 1, Der f 2, Der p 1, Der p 2, Der p 5, Der p 21, and Der p 23, whereas no significant differences were observed for the remaining components (Supplementary Figure S2).

### 3.7 Multivariate analysis of allergen sensitization by PCA

Principal component analysis (PCA) was performed to explore overall sensitization patterns across crustacean and mite allergens. The first two components (PC1 and PC2) accounted for 56% of the total variance (PC1: 35.2%, PC2: 21.2%). PCA revealed a clear clustering of crustacean tropomyosins (Pen m 1, Pan b, Hom g) together with mite tropomyosins (Der p 10, Blo t 10), consistent with their strong cross-reactivity. In contrast, non-tropomyosin allergens such as Der p 23 and Lep d 2 separated along PC2, indicating distinct sensitization trajectories. These results highlight that while pairwise analyses identified specific differences (e.g., higher Der p 23 reactivity in Pen m 1–positive subjects), PCA provides a complementary multivariate perspective, capturing both shared and unique contributions of individual allergens (Supplementary Figure S3).

### 3.8 Relationship between Mite sensitization and severe allergic reactions

**Figure 3:**
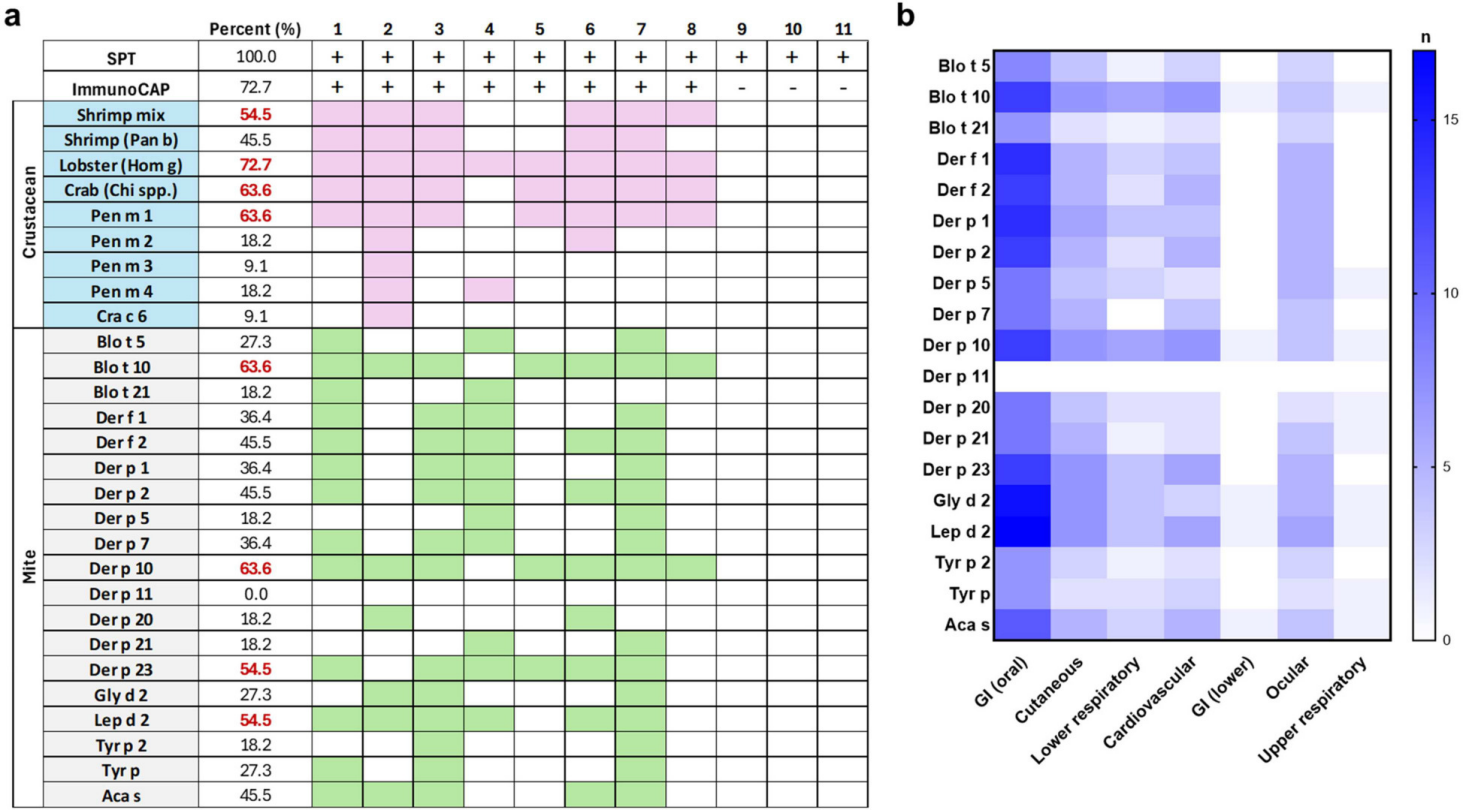
Sensitization profile to mite allergens in crustacean-allergic subjects. **(a)** Sensitization data for 11 subjects who experienced anaphylaxis. Each column represents an individual subject, with positive results indicated by color shading. Percentages represent the proportion of anaphylactic subjects sensitized to each allergen, with values ≥50% highlighted in red. IgE levels ≥0.35 kU/L are shown in pink for crustacean allergens and green for mite allergens. **(b)** Heatmap showing symptom distribution across 42 crustacean-allergic subjects. The intensity of blue shading represents the number of subjects experiencing symptoms in each category.

To assess the relationship between mite sensitization and severe allergic reactions, an analysis was conducted on 11 subjects who experienced anaphylaxis. Sensitization to crustacean allergens was highest for lobster (72.7%) and crab (63.6%), highlighting their potential role in triggering anaphylaxis. Among these individuals, 63.6% were sensitized to Der p 10, followed by 54.5% to Der p 23 and Lep d 2 (Figure 3a).

A heatmap analysis of 42 crustacean-allergic subjects for whom detailed symptom information was available, identified distinct patterns of symptom distribution across various organ systems. Oral gastrointestinal symptoms were the most frequently reported, followed by cutaneous manifestations. Notably, subjects sensitized to Der p 10 and Blo t 10 exhibited a higher prevalence of lower respiratory symptoms. Additionally, sensitization to Blo t 10, Der p 10, Der p 23, and group 2 was highly associated with an increased frequency of cardiovascular symptoms (Figure 3b).

In the subset with complete symptom annotation (*n* = 42), we assessed whether symptom distribution varied across four sIgE classes (0.35–1, 1–5, 5–15, >15 kU/L) for anti-Der p 1, Der p 2, Der p 10, and Der p 23. Symptoms were infrequent at low sIgE (0.35–1 kU/L) but concentrated at higher levels (≥5 kU/L), with oral gastrointestinal manifestations predominating. At higher sIgE, lower-respiratory involvement was more represented for Der p 10 and Der p 23 than for Der p 1 or Der p 2, whereas lower-gastrointestinal and upper-respiratory symptoms remained uncommon across components. Cardiovascular symptoms were largely confined to higher sIgE classes—most evident for Der p 10 and Der p 23, followed by Der p 1 and Der p 2 (Supplementary Figure S4).

### 3.9 Sequence alignment

To explore potential co-sensitization patterns on molecular level, a sequence alignment analysis was conducted using UniProt for allergens with significant IgE reactivity. The results identified notable sequence identity within the tropomyosin and arginine kinase families, suggesting their role in molecular cross-reactivity (Supplementary Figure S5).

In Pen m 1-positive subjects, we observed IgE reactivity to Der p 10 and Blo t 10, which share 80.2% protein sequence identity, reinforcing their structural similarity and subsequent immunological cross-reactivity. However, Der p 23 showed low sequence identity (22.47% with Pen m 1), suggesting a weaker molecular relationship (Supplementary Figure S5a). In Pen m 2-positive subjects, Der p 20 shared 78.65% identity with Pen m 2, indicating a possible association in arginine kinase sensitization. To refine this comparison at the antibody-accessible level, we mapped predicted linear B-cell epitopes across both proteins and observed high conservation across multiple epitope regions using in-silico analysis, with an overall epitope-level identity of ∼71.8%. Notably, two epitopes were fully conserved (10/10 and 8/8 residues), and several showed ≥80% identity (e.g., 19/22, 25/28, 56/71 across merged regions), whereas the least-conserved epitope was 4/8 (50%). Nevertheless, the computational prediction of potential cross-reactivity epitopes need subsequent experimental validation to confirm IgE reactivity at these sites (Supplementary Figure S6).

Lower sequence identities were observed for Gly d 2 (22.31%) and Lep d 2 (19.26%) (Supplementary Figure S5b). For Cra c 6-positive subjects, no high sequence identity was observed with mite allergens. Der p 5 exhibited 23.53% identity, while Der p 23 and Der p 20 shared 13.7% and 15.56% identity, respectively, with Cra c 6 (Supplementary Figure S5c).

### 3.10 Sensitization frequency and IgE reactivity to Der p allergens

Among the Der p allergens analyzed, sensitization was most frequently observed to Der p 23 (61.1%), followed closely by Der p 2 (59.3%) and Der p 1 (57.4%). Sensitization to Der p 10 was lower, occurring in 48.1% of subjects. However, the highest median IgE concentration was observed for Der p 2 (9.0 kU/L). Although Der p 1 showed a high sensitization frequency, the corresponding IgE level was lower than that for both Der p 2 and Der p 23. This pattern suggests that IgE-binding intensity does not always parallel the frequency of sensitization (Supplementary Figure S7).

## 4 Discussion

Shellfish allergy is one of the leading causes of food-induced anaphylaxis worldwide and is particularly prevalent in tropical and subtropical regions. This study provides a detailed molecular characterization of IgE sensitization patterns in a well-characterised cohort of crustacean-allergic individuals in a tropical setting, highlighting the complex interplay between shellfish and house dust mite (HDM) allergens. Our data indicates that both tropomyosin and non-tropomyosin components significantly contribute to allergic sensitization, emphasizing the complex nature of crustacean-HDM cross-reactivity, offering new insights into both expected and novel sensitization pathways. This study provides one of the most detailed molecular assessments to date of mite allergen sensitization in crustacean-allergic individuals to date.

Among the mite allergens assessed, the highest IgE reactivity was observed to Lep d 2 (64.8%) and Der p 23 (61.1%), followed closely by Der f 2, Der p 1, Der p 2, Der f1, and Gly d2. This pattern aligns with prior findings identifying group 1 and 2 allergens as major immunodominant HDM proteins (26–29). These proteins are known to promote allergic inflammation through protease activity (group 1) and innate immune activation via MD-2-related lipid recognition domains (group 2). The prominence of group 2 mite allergens in this cohort may reflect their functional roles in innate immune activation. These allergens belong to the ML (MD-2-related lipid recognition) superfamily and can mimic MD-2, the co-receptor for TLR4, thereby enhancing allergenic potential (30–32). While no ML-domain allergens have yet been identified in crustaceans, the presence of an MD-2-like protein in shrimp (LvML), which binds lipopolysaccharide (LPS), may suggest a distant evolutionary link (33). Though lacking direct IgE cross-reactivity, the shared innate immune activation pathways may contribute to sensitization synergy in co-exposed individuals.

Interestingly, this cohort showed considerably higher IgE levels to Der p 2 than to Der p 1 and Der p 23, despite similar sensitization frequencies to all three allergens. In contrast, a North American cohort reported sensitization rates of 87% for Der p 1, 79% for Der p 2, and 75% for Der p 23, with IgE levels to Der p 23 approximately fourfold lower than those to Der p 1 and Der p 2, and the highest IgE levels observed for Der p 2 (34). Similarly, the MITRA trial reported sensitization rates of 78% for Der p 1, 86% for Der p 2, and 66% for Der p 23, with IgE levels to Der p 23 being 2.5–3.5-fold lower than those to Der p 1 and Der p 2, again with the highest IgE levels observed for Der p 2 (35).

Structural studies on Lep d 2 have shown conserved cysteine motifs that influence T-cell activation (36, 37), and although similar motifs are partially present in LvML, current evidence does not support direct IgE cross-reactivity. However, the significant IgE recognition of mite group 2 allergens in crustacean-allergic individuals points toward potential shared immunostimulatory pathways or atopic predisposition, warranting further investigation.

Tropomyosin (Pen m 1) is a well-established pan-allergen in invertebrates and is recognized for its high cross-reactivity across crustaceans, mites, cockroaches, and other arthropods (13, 14, 24, 38, 39). In this study, although overall median IgE levels to Der p 10 were low, subjects sensitized to Pen m 1 exhibited significantly higher IgE levels to Der p 10 and Blo t 10 compared with Pen m 1-negative individuals. This observation aligns with the high amino acid sequence identity among tropomyosins from crustaceans and mites and subsequent IgE binding (17, 18, 24, 26, 38–40). In contrast, much lower IgE recognition of Der p 5, Der p 7, and Der p 11 was observed in our cohort, which may reflect geographic variation in exposure or immune recognition.

The high sensitization rates to several mite allergens in this cohort confirms the hypothesis that in tropical climates, where mite exposure is perennial and intense, polysensitization to multiple mite components is common among individuals with crustacean allergy. Importantly, our current finding suggest a complex sensitization profile extending beyond the undisputed tropomyosin-mediated cross-reactivity. This broader complexity was supported by PCA, which provided a complementary multivariate perspective, demonstrating clustering of crustacean and mite tropomyosins while distinguishing non-tropomyosin allergens along PC2.

Elevated IgE levels to Der p 20 were observed among Pen m 2–positive individuals, indicating a related IgE sensitization pattern. Sequence identity between these allergens (>78%) supports true molecular cross-reactivity, consistent with earlier work identifying arginine kinase as an important pan-allergen in invertebrates (38, 41). Additional significant responses to Gly d 2 and Lep d 2 may reflect independent sensitization events or yet unidentified structural similarities.

For Cra c 6-positive individuals (sensitized to crustacean troponin C), significant IgE responses were observed to Der p 5, Der p 20, Der p 23, Gly d 2, Lep d 2, and Tyr p 2. However, low sequence identity between Cra c 6 and these mite allergens suggests that these IgE responses may arise from recognition of structurally similar motifs rather than shared linear epitopes, although further structural and functional studies are needed to confirm this.

One additional mite allergen is also deemed to be of importance in the context of co-reactivity and clinical relevance. Der p 23, a recently identified major allergen associated with the peritrophic matrix of mite faecal pellets, was also significantly elevated in Pen m 1-positive subjects, despite having low amino acid sequence identity with tropomyosin. Reported sensitization rates to Der p 23 vary widely across populations, with frequencies of 64% in this study's crustacean-allergic cohort, 35% in Austria, and up to 79% in atopic subjects from Hong Kong. While these Figures are not directly comparable due to differences in study populations and sensitization profiles, they nevertheless highlight geographic variability that may reflect climatic influences, particularly in warm, humid environments where mites thrive (28, 42, 43). In addition, studies have reported that sensitization to Der p 23 is linked to severe allergic rhinitis and asthma symptoms in individuals with mite allergy (44, 45). A recent study from Singapore analysing IgE binding on Der p 23 using site-directed mutagenesis identified two residues, K44 and E46 located in the N-terminal region, as key antibody binding sites. The study also noted that E84, located on the opposite surface, may enable dual IgE binding and cross-linking. Subsequently, more severe allergic rhinitis was associated with higher numbers of IgE binding residues (46). This suggests co-reactivity in our population rather than classic cross-reactivity and raises the possibility of multi-epitope sensitization or epitope spreading. Supporting this, emerging evidence has demonstrated the presence of IgE antibodies that recognize possible conformational epitopes across unrelated protein families (47).

Recent population-based evidence has shown that shrimp-specific IgE is associated with increased cardiovascular mortality, indicating that food allergen sensitization may have systemic effects beyond classical allergic manifestations (48). Such findings highlight that IgE-mediated responses, whether triggered by food or environmental allergens, can have widespread physiological consequences. The analysis of a subgroup of 11 individuals who experienced anaphylaxis revealed disproportionately high rates of sensitization to Der p 10, Der p 23, and Lep d 2, suggesting that these mite allergens may act as risk markers for severe allergic reactions. The elevated rates of lower respiratory symptoms in individuals sensitized to tropomyosin may reflect its role as a cross-reactive pan-allergen between mites and crustaceans (49), as shown clinically by the association of IgE to shrimp tropomyosin (Pen a 1) and its mite homolog Der p 10 with asthma and respiratory manifestations, and experimentally by murine models in which shrimp tropomyosin induced eosinophilic airway inflammation and asthma-like Th2 responses (50, 51). Furthermore, the strong association between Der p 23 sensitization and systemic symptoms reinforces its emerging status as a clinically relevant HDM allergen. The wide range of symptoms involving multiple organ systems in highly sensitized individuals supports the importance of molecular profiling in stratifying allergy severity and specific management strategies.

The role of HDM immunotherapy should also be considered when interpreting IgE profiles. In our study, treated individuals showed higher IgE levels to several major mite allergens, though without baseline data it is unclear if this reflects pre-existing sensitization or therapy effects. These differences were not uniform across allergens.

Our findings also highlight the diagnostic implications of advanced multiplex testing. A subset of participants exhibited positive SPT and/or ImmunoCAP responses but were negative for all measured crustacean components and extracts in the ALEX2 panel. Within this crustacean-negative subgroup, IgE reactivity was nevertheless detected against several major mite allergens, most prominently Der p 1 (50%) and Der p 2 (42.9%), with additional responses to Der p 23 (21.4%) and Der p 10 (14.3%). This selective pattern suggests that, in some individuals, shellfish allergy may reflect primary sensitization to mite allergens with clinical cross-reactivity, whereas in others it may point to the existence of as yet unidentified IgE-binding epitopes in crustaceans not captured by the current component panel. These results emphasize the diagnostic limitations of conventional single-extract testing and reinforce the value of component-resolved diagnostics (CRD) in resolving complex sensitization profiles.

The current study represents one of the most detailed molecular evaluations of mite allergen sensitization among individuals with crustacean allergy conducted to date. The findings highlight not only the well-characterized tropomyosin-mediated cross-reactivity but also emerging roles for arginine kinase, troponin C, and group 2 allergens, and particularly Der p 23, in driving complex sensitization patterns.

This study emphasizes that shellfish allergy in tropical regions is influenced not only by primary sensitization to crustacean proteins but also by complex interactions with HDM allergens, potentially involving both shared IgE recognition and concurrent sensitization. These insights emphasize the need for refined diagnostic approaches that incorporate component-resolved analysis to distinguish true cross-reactivity from co-sensitization. Future studies should explore the functional relevance of these sensitization profiles in diverse populations and examine the predictive value of key mite allergens in assessing the risk of severe allergic reactions. Ultimately, these findings support a personalized approach to allergy diagnosis and management in the era of molecular allergology.

## 5 Limitations and future directions

While this study provides novel insights, some limitations should be noted. The cohort size was modest, which may reduce the power of subgroup analyses. In addition, information on IgE levels prior to HDM immunotherapy was unavailable, and functional cross-reactivity was not directly confirmed by inhibition or cellular assays.

Future research should aim to expand the cohort and incorporate inhibition or cell-based assays to verify cross-reactivity. These refinements will help strengthen the clinical interpretation of sensitization patterns and enhance the utility of component-resolved diagnostics in food and environmental allergy.

## 6 Conclusion

In summary, this study provides new insights into the complex molecular sensitization patterns in crustacean-allergic individuals living in tropical environments. Beyond the well-established role of tropomyosin, our findings highlight significant contributions from non-tropomyosin allergens, particularly arginine kinase, Der p 23, and Lep d 2, in shaping IgE responses. The overlap between crustacean and mite allergens reflects both cross-reactive and independent sensitization mechanisms cross-reactive and independent sensitization mechanisms, with important implications for clinical symptoms and risk stratification. These results highlight the diagnostic value of component-resolved testing and the need for a personalized, molecular-based approach to allergy assessment and management.

## Data Availability

The original contributions presented in the study are included in the article/Supplementary Material, further inquiries can be directed to the corresponding author.
