# Supplementary material for: Allergic sensitization in the tropics: unravelling co-sensitization between crustacean and mites
Source: Front Allergy. 2025 Nov 14;6:1674423. doi: 10.3389/falgy.2025.1674423 (PMC12660283; doi:10.3389/falgy.2025.1674423)
Supplement: Supplementary file 1 [file Datasheet1.pdf]

Supplementary Material

1 Supplementary Figures and Tables

1.1 Supplementary Table 1

Supplementary Table 1: Crustacean and mite allergens in the ALEX2 assay with allergen codes, names, and biochemical classifications.

	Component / Extract	Allergen-code	Common name	Scientific name	Component	Biochemical designation
Crustacean	E	f23	Crab	*Chionoecetes spp.*		
	E	f80	Lobster	*Homarus gammarus*		
	E	f515	Northern prawn	*Pandalus borealis*		
	E	f24	Shrimp mix	*Litopaenaeus setiferus, Farfantepenaeus aztecus,Farfantepenaeus dourarum*		
	C	f517	Black tiger shrimp	*Penaeus monodon*	rPen m 1	Tropomyosin
	C	f545	Black tiger shrimp	*Penaeus monodon*	rPen m 2	Arginine Kinase
	C	f552	Black tiger shrimp	*Penaeus monodon*	rPen m 3	Myosin light chain
	C	f524	Black tiger shrimp	*Penaeus monodon*	rPen m 4	Sarcoplasmic Calcium Binding Protein
	C	f529	Brown shrimp	*Crangon crangon*	rCra c 6	Troponin C
Mite	E	d72	Tyrophagus putrescentiae	*Tyrophagus putrescentiae*		
	E	d70	Acarus siro	*Acarus siro*		
	C	d100	American house dust mite	*Dermatophagoides farinae*	rDer f 1	Cysteine Protease
	C	d101	American house dust mite	*Dermatophagoides farinae*	rDer f 2	NPC2 Family
	C	d300	Blomia tropicalis	*Blomia tropicalis*	rBlo t 5	Mite, Group 5
	C	d301	Blomia tropicalis	*Blomia tropicalis*	rBlo t 10	Tropomyosin
	C	d302	Blomia tropicalis	*Blomia tropicalis*	rBlo t 21	Unknown
	C	d202	European house dust mite	*Dermatophagoides pteronyssinus*	rDer p 1	Cysteine Protease
	C	d203	European house dust mite	*Dermatophagoides pteronyssinus*	rDer p 2	NPC2 Family
	C	d103	European house dust mite	*Dermatophagoides pteronyssinus*	rDer p 5	Unknown
	C	d104	European house dust mite	*Dermatophagoides pteronyssinus*	rDer p 7	Mite Group 7
	C	d205	European house dust mite	*Dermatophagoides pteronyssinus*	rDer p 10	Tropomyosin
	C	d102	European house dust mite	*Dermatophagoides pteronyssinus*	rDer p 11	Myosin, heavy chain
	C	d303	European house dust mite	*Dermatophagoides pteronyssinus*	rDer p 20	Arginine Kinase
	C	d304	European house dust mite	*Dermatophagoides pteronyssinus*	rDer p 21	Unknown
	C	d209	European house dust mite	*Dermatophagoides pteronyssinus*	rDer p 23	Peritrophin-like protein domain
	C	d105	Glycyphagus domesticus	*Glycyphagus domesticus*	rGly d 2	NPC2 Family
	C	d305	Lepidoglyphus destructor	*Lepidoglyphus destructor*	rLep d 2	NPC2 Family
	C	d306	Tyrophagus putrescentiae	*Tyrophagus putrescentiae*	rTyr p 2	NPC2 Family

1.2 Supplementary Figure 1

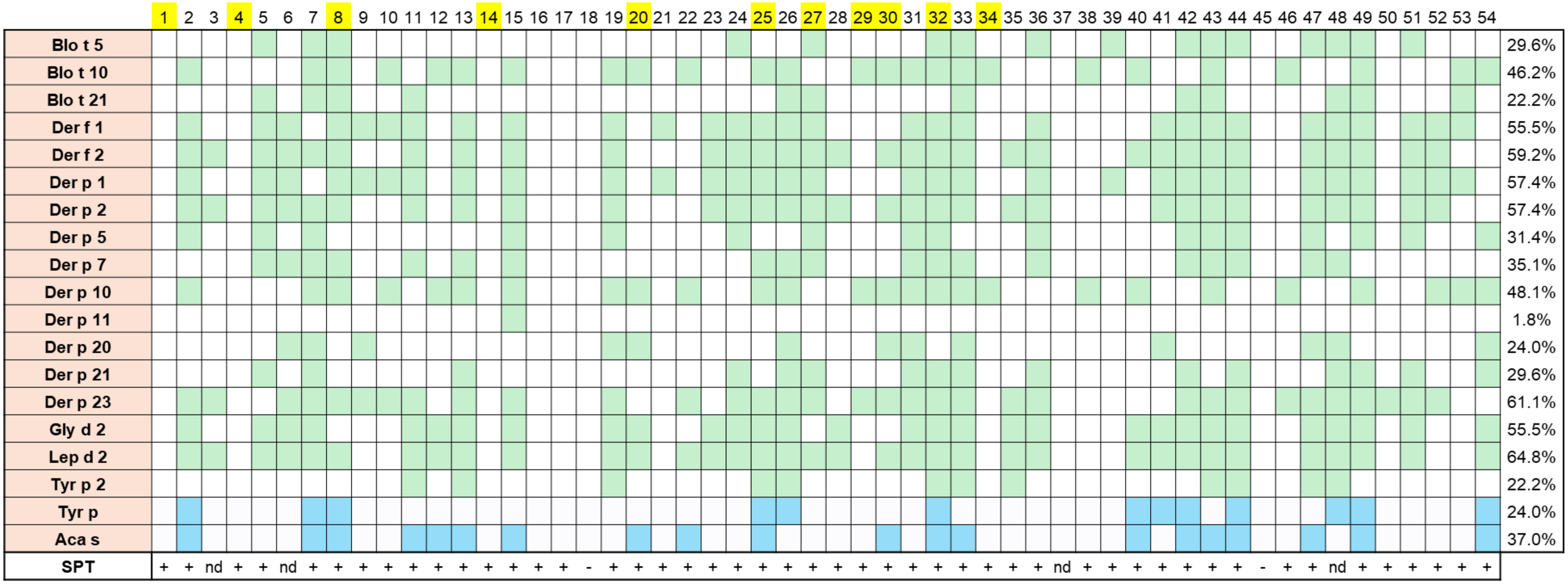

Supplementary Figure 1: Sensitization profile of 54 crustacean-allergic subjects to mite allergens based on serological and skin prick test (SPT) results. Positive test results are highlighted in color, with corresponding frequencies indicated in the right panel. Green shading represents positive IgE responses to mite single components, while blue shading indicates positive responses to mite whole extract. Numbers highlighted in yellow indicate individuals who experienced anaphylaxis. A specific IgE result of ≥0.35 kU/L is considered positive. SPT: Skin Prick Test; nd: not determined.

1.3 Supplementary Table 2

Supplementary Table 2: Frequency of IgE sensitization to individual mite allergens among 14 subjects negative to all crustacean components.

Allergen name	Positive (n)	Positive %
**Blo t 5**	4	28.6
**Blo t 10**	1	7.1
**Blo t 21**	3	21.4
**Der f 1**	6	42.9
**Der f 2**	6	42.9
**Der p 1**	7	50
**Der p 2**	6	42.9
**Der p 5**	3	21.4
**Der p 7**	2	14.29
**Der p 10**	2	14.3
**Der p 11**	0	0
**Der p 20**	0	0
**Der p 21**	3	21.4
**Der p 23**	3	21.4
**Gly d 2**	5	35.7
**Lep d 2**	5	35.7
**Tyr p 2**	0	0
**Tyr p**	1	7.1
**Aca s**	1	7.1

1.4 Supplementary Figure 2

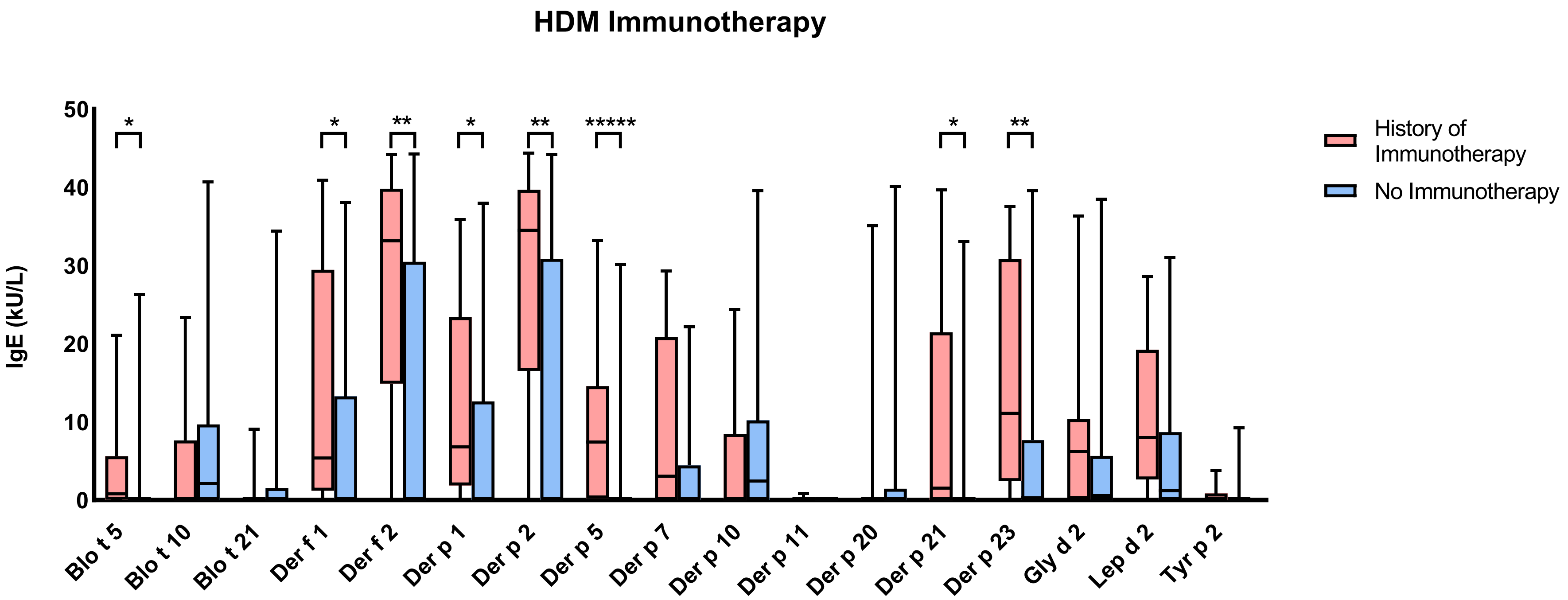

Supplementary Figure 2: Comparison of mite-specific IgE (kU/L) between subjects with (n = 16) and without (n = 38) a history of HDM immunotherapy among the 54 crustacean-allergic individuals. Mite-specific IgE levels are shown for individual allergens, with bars representing median and interquartile range. Statistical significance between groups is indicated above the comparisons (*p < 0.05; **p < 0.01; ***p < 0.001).

1.5 Supplementary Figure 3

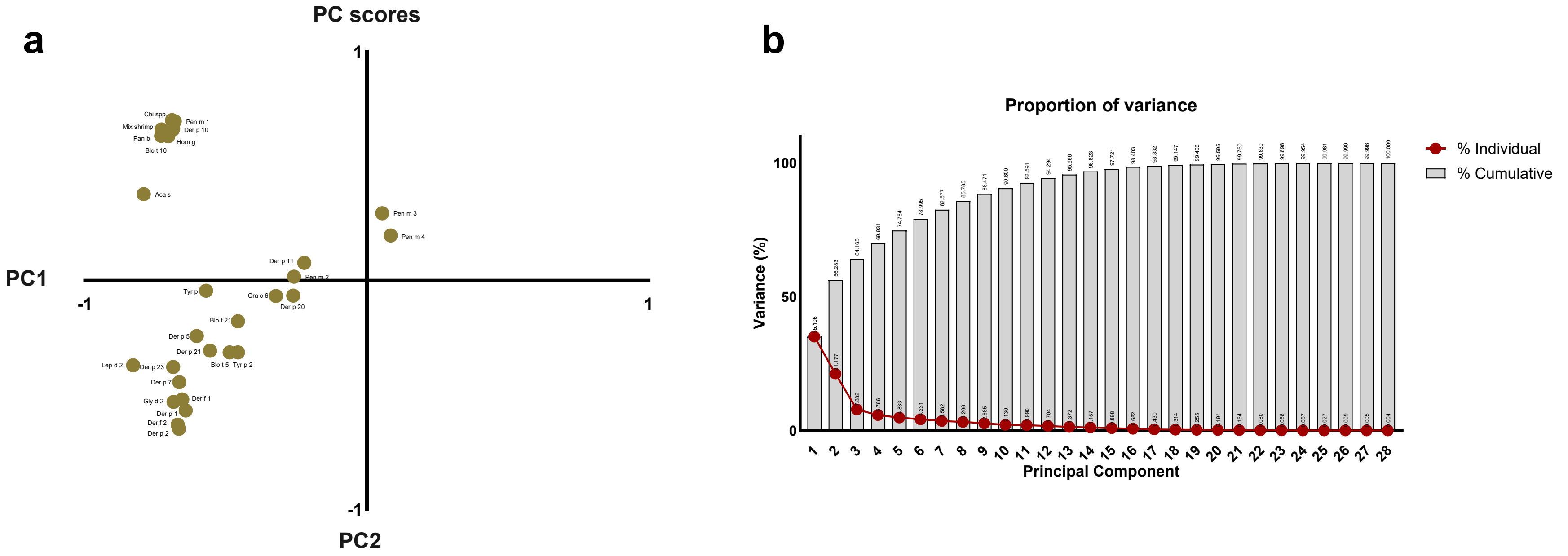

Supplementary Figure 3. PCA of IgE sensitization to crustacean and mite allergens. (a) Score plot of the first two principal components (PC1 and PC2). (b) Proportion of variance explained by each component, showing individual and cumulative variance.

1.6 Supplementary Figure 4

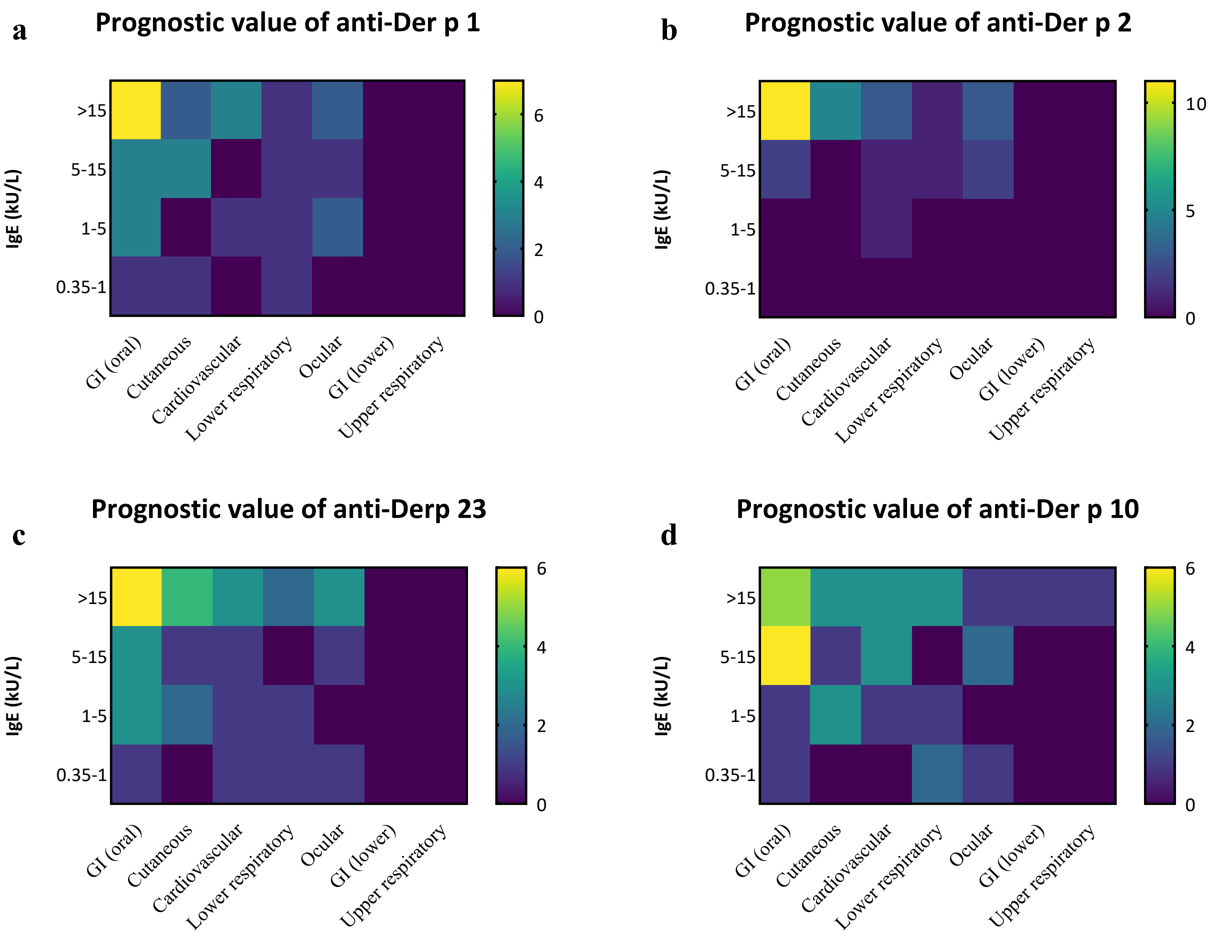

Supplementary Figure 4. Prognostic value of mite-specific IgE levels in relation to clinical symptoms among crustacean-allergic subjects. Heatmaps show IgE levels (kU/L) to (a) Der p 1, (b) Der p 2, (c) Der p 23, and (d) Der p 10 across symptom categories (gastrointestinal, cutaneous, cardiovascular, lower respiratory, ocular, and upper respiratory). Color intensity indicates the number of subjects in each IgE class (0.35–1, 1–5, 5–15, >15 kU/L) reporting the respective symptoms.

1.7 Supplementary Figure 5

a

			Pen m 1	Der p 23	Blo t 10	Der p 10
1	tr|E1A682|E1A682_PENMO	Pen m 1	100			
2	sp|L7N6F8|DEP23_DERPT	Der p 23	22.47	100		
3	tr|A7XZI4|A7XZI4_BLOTA	Blo t 10	80.21	26.97	100	
4	sp|O18416|TPM_DERPT	Der p 10	80.21	24.72	94.37	100

b

			Pen m 2	Der p 20	Gly d 2	Lep d 2
1	sp|C7E3T4|KARG_PENMO	Pen m 2	100			
2	tr|B2ZSY4|B2ZSY4_DERPT	Der p 20	78.65	100		
3	sp|Q9U5P7|ALL21_GLYDO	Gly d 2	23.14	22.31	100	
4	sp|P80384|ALL2_LEPDS	Lep d 2	19.26	19.26	56	100

c

			Cra c 6	Der p 5	Der p 23	Der p 20	Tyr p 2	Gly d 2	Lep d 2
1	tr|D7F1Q2|D7F1Q2_CRACN	Cra c 6	100						
2	sp|P14004|ALL5_DERPT	Der p 5	23.53	100					
3	sp|L7N6F8|DEP23_DERPT	Der p 23	13.7	22.06	100				
4	tr|B2ZSY4|B2ZSY4_DERPT	Der p 20	15.56	17.09	19.54	100			
5	sp|O02380|ALL2_TYRPU	Tyr p 2	7.46	18.57	20.41	21.43	100		
6	sp|Q9U5P7|ALL21_GLYDO	Gly d 2	7.41	9.09	8.82	19.84	44.44	100	
7	sp|P80384|ALL2_LEPDS	Lep d 2	10.29	21.74	20.41	16.43	47.14	56	100

Supplementary Figure 5: Percent identity matrices for selected allergens based on sequence alignment. The matrices represent the percentage of sequence identity between allergens, with higher identity values highlighted in pink. (a) Percent identity matrix for Pen m 1, Der p 10, Blo t 10, and Der p 23, showing strong sequence similarity between Blo t 10 and Der p 10 (94.37%) and between Blo t 10 and Pen m 1 (80.21%). (b) Percent identity matrix for Pen m 2, Der p 20, Gly d 2, and Lep d 2, highlighting a significant similarity between Der p 20 and Pen m 2 (78.65%). (c) Percent identity matrix for Cra c 6, Der p 5, Der p 23, Der p 20, Tyr p 2, Gly d 2, and Lep d 2, showing varying degrees of sequence identity, with the highest similarity observed between Lep d 2 and Gly d 2 (56%).

1.8 Supplementary Figure 6

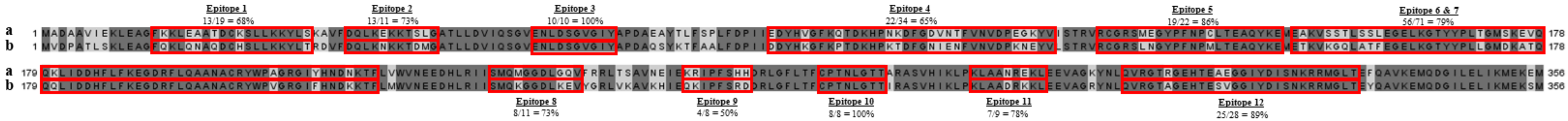

Arginine Kinase:

a) AAO15713.1/1-356: Penaeus monodon (Pen m 2)

b) ACD50950.1/1-356: Dermatophagoides pteronyssinus (Der p 20)

Overall Epitope Similarity: **71.75%**

Supplementary Figure 6: Sequence alignment of arginine kinase from *Penaeus monodon* (Pen m 2; AAO15713.1) and *Dermatophagoides pteronyssinus* (Der p 20; ACD50950.1), with predicted linear B-cell epitopes highlighted in red boxes. Overall epitope similarity was 71.75%.

1.9 Supplementary Figure 7

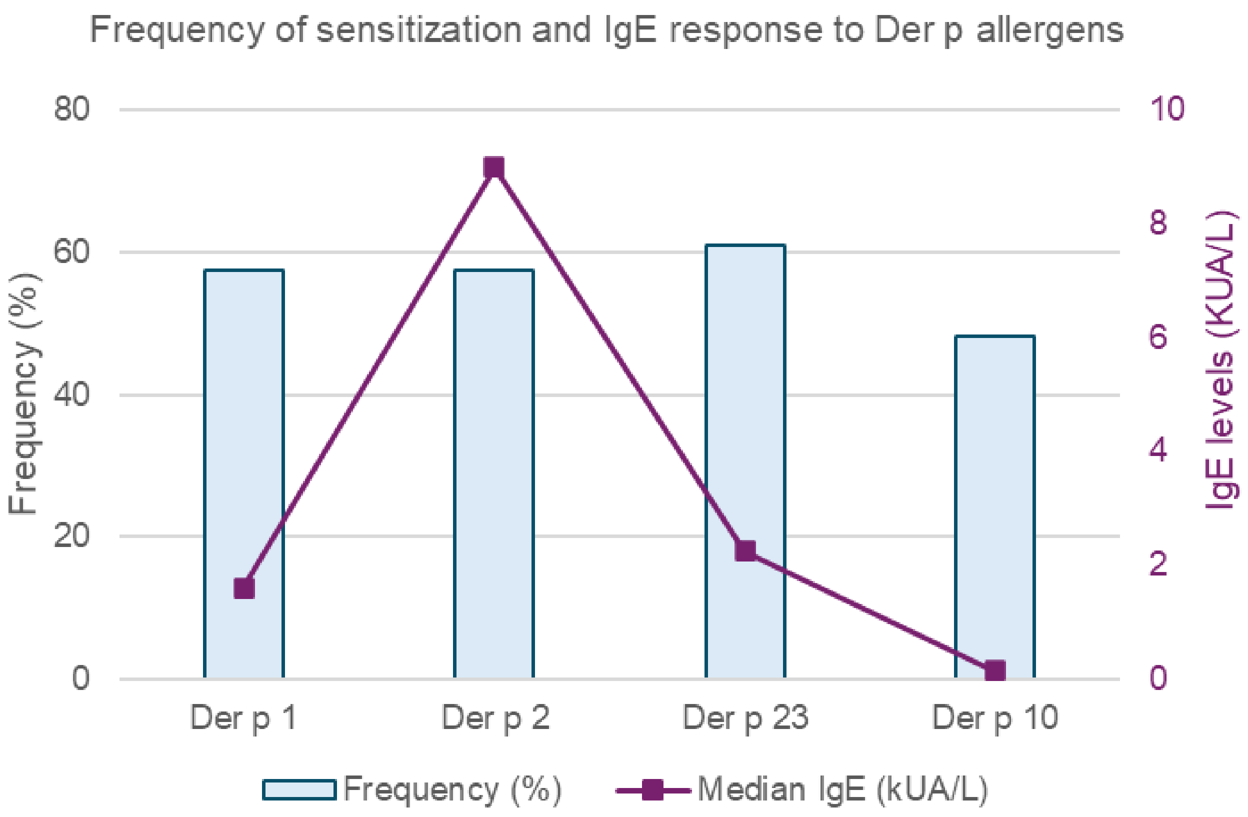

Supplementary Figure 7. Frequency of sensitization and allergen-specific IgE levels to *Der* p allergens. Bars represent the proportion of subjects sensitized to *Der* p 1, *Der* p 2, *Der* p 23, and *Der* p 10, expressed as percentage frequency. Purple squares indicate the median allergen-specific IgE levels (kUA/L) for each allergen,
